# Investigation of Visual Stimulus With Various Colors and the Layout for the Oddball Paradigm in Evoked Related Potential-Based Brain–Computer Interface

**DOI:** 10.3389/fncom.2019.00024

**Published:** 2019-04-26

**Authors:** Miaoji Guo, Jing Jin, Yong Jiao, Xingyu Wang, Andrzej Cichockia

**Affiliations:** ^1^Key Laboratory of Advanced Control and Optimization for Chemical Processes, Ministry of Education, East China University of Science and Technology, Shanghai, China; ^2^Skolkowo Institute of Science and Technology (SKOLTECH), Moscow, Russia; ^3^Systems Research Institute PAS, Warsaw, Poland; ^4^Department of Informatics, Nicolaus Copernicus University (UMK), Torun, Poland

**Keywords:** brain–computer interface, ERP, color of stimulus, visual stimulus, single character paradigm

## Abstract

**Objective:** Stimulus visual patterns, such as size, content, color, luminosity, and interval, play key roles for brain–computer interface (BCI) performance. However, the three primary colors to be intercompared as a single variable or factor on the same platform are poorly studied. In this work, we configured the visual stimulus patterns with red, green, and blue operating on a newly designed layout of the flash pattern of BCI to study the waveforms and performance of the evoked related potential (ERP).

**Approach:** Twelve subjects participated in our experiment, and each subject was required to finish three different color sub-experiments. Four blocks of the interface were presented along the edge of the screen, and the other four were assembled in the center, aiming to investigate the problem of adjacency distraction. Repeated-measures ANOVA and Bonferroni correction were applied for statistical analysis.

**Main results:** The averaged online accuracy was 98.44% for the red paradigm, higher than 92.71% for the green paradigm, and 93.23% for the blue paradigm. Furthermore, significant differences in online accuracy (*p* < 0.05) and information transfer rate (*p* < 0.05) were found between the red and green paradigms.

**Significance:** The red stimulus paradigm yielded the best performance. The proposed design of ERP-based BCI was practical and effective for many potential applications.

## Introduction

Brain–computer interface (BCI) enables patients suffering from movement disorders to communicate with others or interact with the outside world through electroencephalogram (EEG), magnetoencephalography (MEG), functional magnetic resonance imaging (fMRI), and more (Vidal, 1973, 1977; Wolpaw et al., 2000, 2002). Evoked related potential (ERP) from EEG/MEG can be reliably measured by scalp electrodes or sensors (Sutton et al., 1965; Coles and Rugg, 1995). To date, most research works on BCI can be roughly divided into several categories according to the types of signals used, especially ERP-based BCI (Farwell and Donchin, 1988; Furdea et al., 2009; Kübler et al., 2009; Zhang et al., 2012; including P300-BCI), motor imagery BCI (Pfurtscheller and Neuper, 2001; Wang et al., 2006; Hwang et al., 2009; Jiao et al., 2018), steady-state visual evoked potentials (SSVEP) BCI (Ortner et al., 2011; Jiao et al., 2016; Nakanishi et al., 2018; Zhang et al., 2018), hybrid BCI (Pfurtscheller et al., 2010; Li et al., 2016), and so on. In the present work, we focused on ERP-based BCI, which is one of the most promising approaches.

A commonly used component in ERP is the visual evoked potential (VEP) P300 or P3, which is generally elicited by the oddball paradigm. P300 is characterized by a latency of 250–500 ms after stimulus, and the positive deflection is stronger than other components (e.g., P100, N170, and N200) prior to it (Sutton et al., 1965). Therefore, the VEP P300-BCI stands for the utilization of P300 as the way to discriminate the target and the non-target. The first VEP P300-BCI, otherwise known as P300 speller, was introduced by Farwell and Donchin (1988). In their study, subjects were asked to sit in front of a screen with a 6 × 6 matrix presenting 26 letters and 10 digits and required to count the number of flashes of target characters silently in the row–column paradigm (RCP). However, adjacency-distraction errors and double-flash errors are the main defects of RCP. To decrease the impact of these two, researchers found ways to address this problem from multiple levels. Townsend et al. (2010) designed an 8 × 9 checkerboard paradigm (CBP) to separate two 6 × 6 matrices and arrange all rows of one matrix to flash randomly first before the columns, thereby effectively avoiding both abovementioned errors. Jin et al. (2010) composed a new method that mathematically combined the stimuli presented to improve the performance and yielded a higher bit rate than that of the RCP. Paralleling with RCP, the single-character paradigm (SCP), in which each character is individually highlighted, fully capable of avoiding adjacency distraction, has also been extensively studied (Fazel-Rezai et al., 2012; Jin et al., 2015). To compare these two mainstream paradigms (RCP and SCP) fairly, Guger et al. invited 100 healthy subjects to perform a spelling task, and the result showed that 72.8% (*N* = 81) of the subjects spelled RCP with 100% accuracy and 55.3% (*N* = 38) of the subjects did the same in SCP. However, the averaged P300 response at Cz for RCP was 7.9 μV lower than the 8.8 μV achieved in SCP (Guger et al., 2009). Moreover, a modified SCP called lateral SCP provided a better performance than RCP with respect to online accuracy and bit rate (Pires et al., 2012). Thus, RCP and SCP are both promising methods to establish a practical BCI system.

The effects brought by stimuli have been explored in many aspects, such as the interstimulus interval (Sellers et al., 2006), the background color of stimulus (Salvaris and Sepulveda, 2009), the face stimulus (Zhang et al., 2012; Jin et al., 2014), the moving stimulus like vertical moving bars (Hong et al., 2009), flipping characters (Martens et al., 2009), zooming symbols (Cheng et al., 2018), and so on. As for color, white and black backgrounds were compared. Consequently, white background was superior to the black one in terms of performance (Salvaris and Sepulveda, 2009). Green (onset)/blue (offset) stimulus yielded a better practical performance in P300-BCI than white/gray stimulus (Takano et al., 2009). Moreover, the luminosity contrast was also investigated for P300 speller (Li et al., 2014). The RGB colors acting as stimuli have been utilized to compare EEG classification algorithms or feature extraction methods (Rasheed and Marini, 2015; Alharbi et al., 2016). However, the paradigm was limited to one square pattern responsible for presenting colors under a gray background, with a stimulus duration of 3 s one time, instead of the oddball paradigm.

In this study, we introduced a new layout of flash pattern on the basis of SCP, with red, green, and blue stimuli under a white background. In addition, aside from P300, other visual ERP waveforms, such as P200 (P2), N2, and N400 (N4), have already been proven beneficial to improve BCI performance. For example, Guo et al. (2008) introduced motion-onset VEPs including P2 and N2, to deliver control command successfully; Jin et al. (2014) suggested that N4 helps improve the online accuracy of ERP-based BCI. Therefore, the waveform features of P2, N2, P3, and N4 were also considered during ERP analysis in our study.

## Materials and Methods

### Subjects

Twelve healthy subjects (S1–S12), comprising six males and six females aged 22–28 years, participated in our experiments. All subjects had normal color vision, and seven of them participated in a BCI experiment for the first time. The local ethics committee approved the consent form and the experimental procedure before any of the subjects participated. All subjects were informed of the whole online-and-offline procedure beforehand, and they were allowed to leave the experiment anytime if they felt uncomfortable during the experiment.

### Experimental Design

A 20-in. LCD, Lenovo UOAFG989, was set with sRGB color gamut and 1,600 × 900 resolution, and its maximum luminous intensity was 200 cd/m^2^ when displaying white. A subject was seated 70 cm away from the display in a dimly lit laboratory, with ambient light of 40 ± 9.2 lx. Psychotoolbox from MATLAB was operated for the flash pattern. Red (255, 117, 117), green (117, 255, 117), and blue (117, 117, 255) colors were chosen to be the stimuli by turns. The stimulus onset asynchrony (SOA) was set to 400 ms, and the duration of stimulus was 200 ms throughout all experiments.

**Figure 1:**
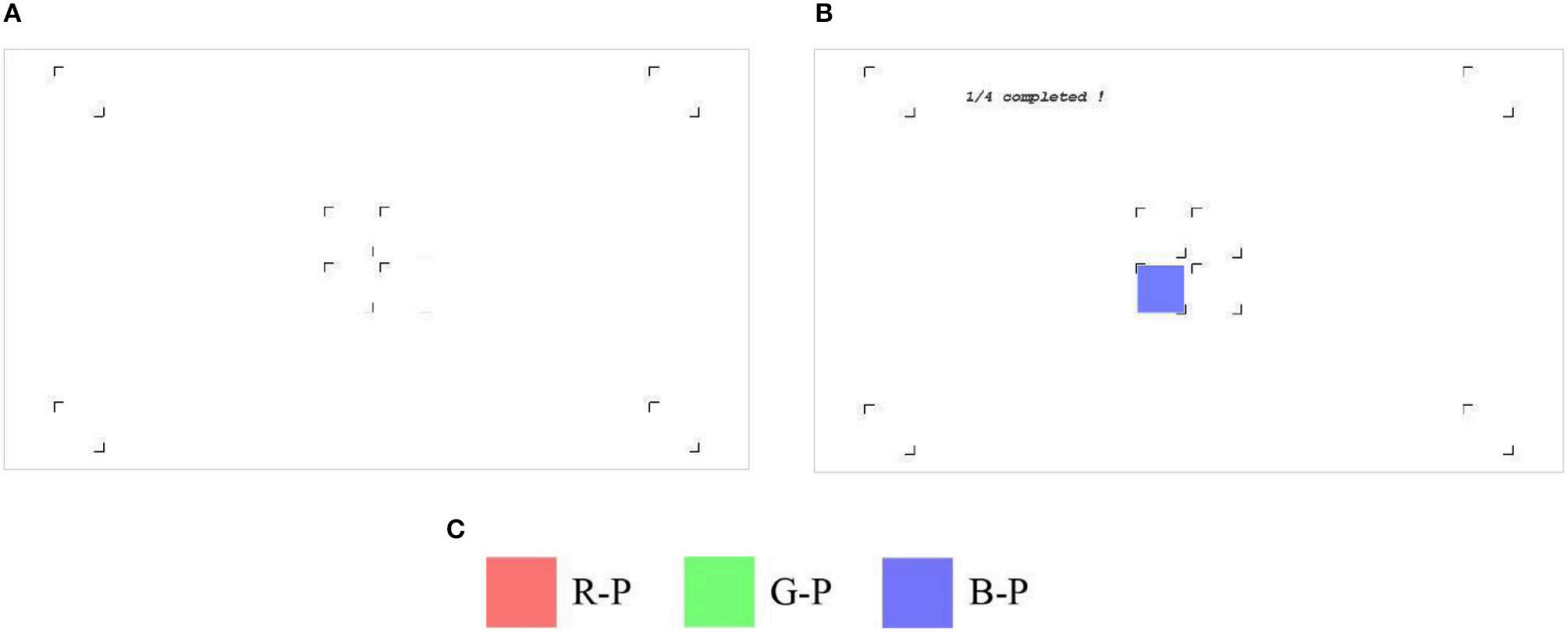
The layout of the experiment. **(A)** The original pattern; **(B)** the screenshot of the “blue” experiment; **(C)** the legend of the three stimuli.

The specific layout of the pattern is shown Figure 1. Four square blocks (108 × 108) were distributed at the four corners of the screen, whereas the other four were assembled in the center. Altogether, eight square blocks took turns to be the target. Figure 1A illustrates the original presentation of the pattern before the experiment began, and Figure 1B was merely captured as an example for the ongoing “blue” experiment. Here, the color of the stimulus can also be represented by red or green in their own color sub-experiment. Figure 1C demonstrates the color configuration of the three paradigms. In this study, three paradigms were presented to every subject in order. We called them R-P (red paradigm), G-P (green paradigm), and B-P (blue paradigm) for convenience.

**Figure 2:**
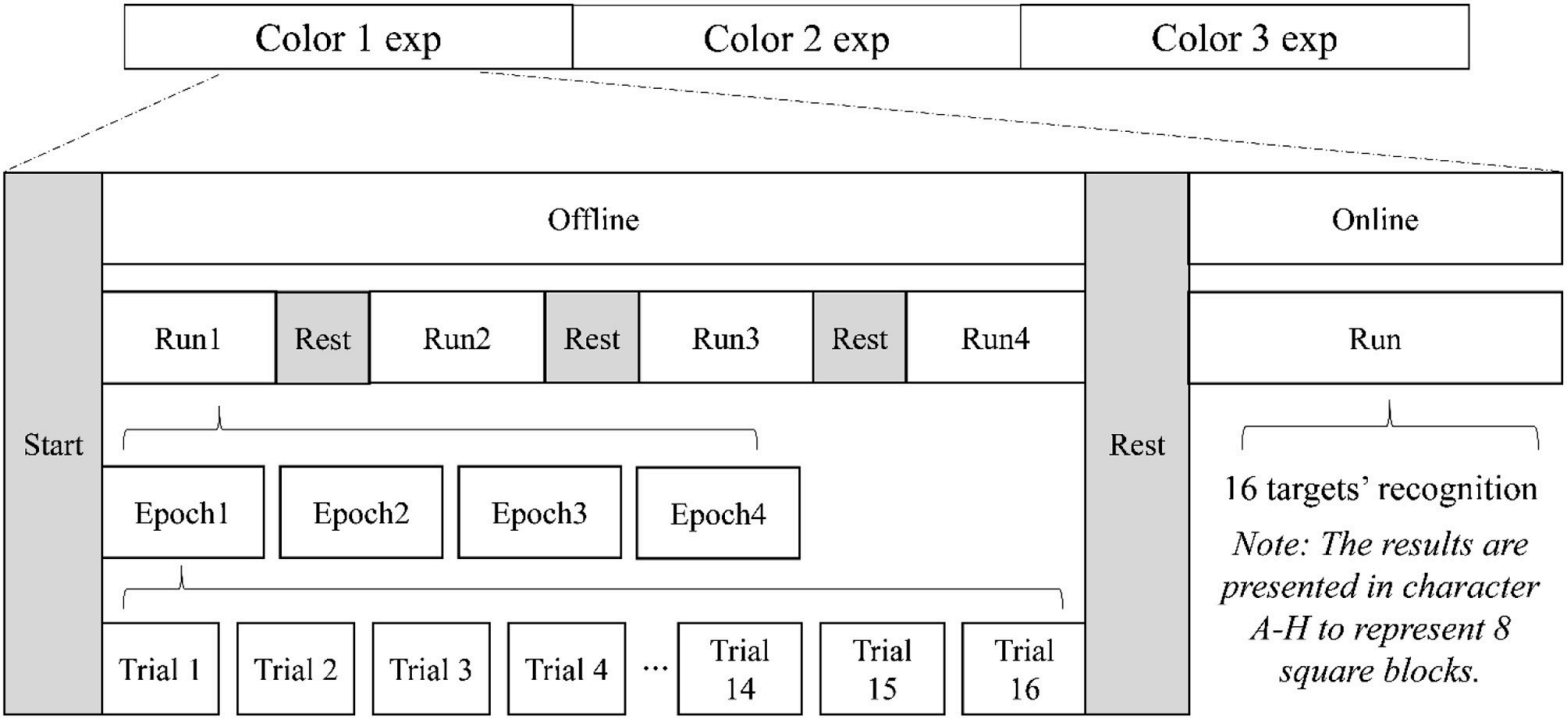
Flowchart of our experiments (note: exp here stands for experiment).

The flowchart of the set of experiments is shown in Figure 2. Each paradigm consisted of offline and online sessions. As illustrated in Figure 2, one offline experiment had four runs, and each run included four epochs. One epoch stood for one target block to be focused on, and 16 trials represented the repeating times applied in each epoch. When an epoch began, the subject focused on the target block where the hint showed before and counted the flashes in the target block silently, at the same time, ignoring other flashes lighted in non-target blocks. When the target flash had been shown for the predetermined (i.e., 16 in this study) times, one epoch finished, and the hint would move on to the next block. Then, that block would take the place of the former as the new target to be focused on. After finishing four runs of offline, a model of the subject would be built. Then, the online experiment would operate 16 blocks to be the targets one by one with feedbacks. As for the feedback, the four blocks near the edge were represented by A (up left), B (up right), C (bottom left), and D (bottom right), and the four in the center with E, F, G, and H were assigned in the same way. The number of trials for recognition was chosen automatically via an adaptive strategy, which was explained in the section Online Strategy (Jin et al., 2011). Compared with offline experiments, the online one saved trials and delivered feedbacks in a timely manner.

Given that the order of “color” displayed could influence the BCI performance, we arranged S1, S2, S9, and S12 to follow the order of R(red)–G(green)–B(blue). S3, S4, S6, and S8 were arranged in G–B–R. S5, S7, S10, and S11 were arranged in B–R–G (see Table 1). This arrangement could lead to relative fairness in the subsequent analysis.

**Table 1:** The order of paradigms for each subject.

	**S1**	**S2**	**S3**	**S4**	**S5**	**S6**	**S7**	**S8**	**S9**	**S10**	**S11**	**S12**
R-P	1	1	3	3	2	3	2	3	1	2	2	1
B-P	2	2	1	1	3	1	3	1	2	3	3	2
G-P	3	3	2	2	1	2	1	2	3	1	1	3

*“1” means the subject did the corresponding paradigm first, and “2” represents second and “3” did the last*.

### Electroencephalogram Acquisition

**Figure 3:**
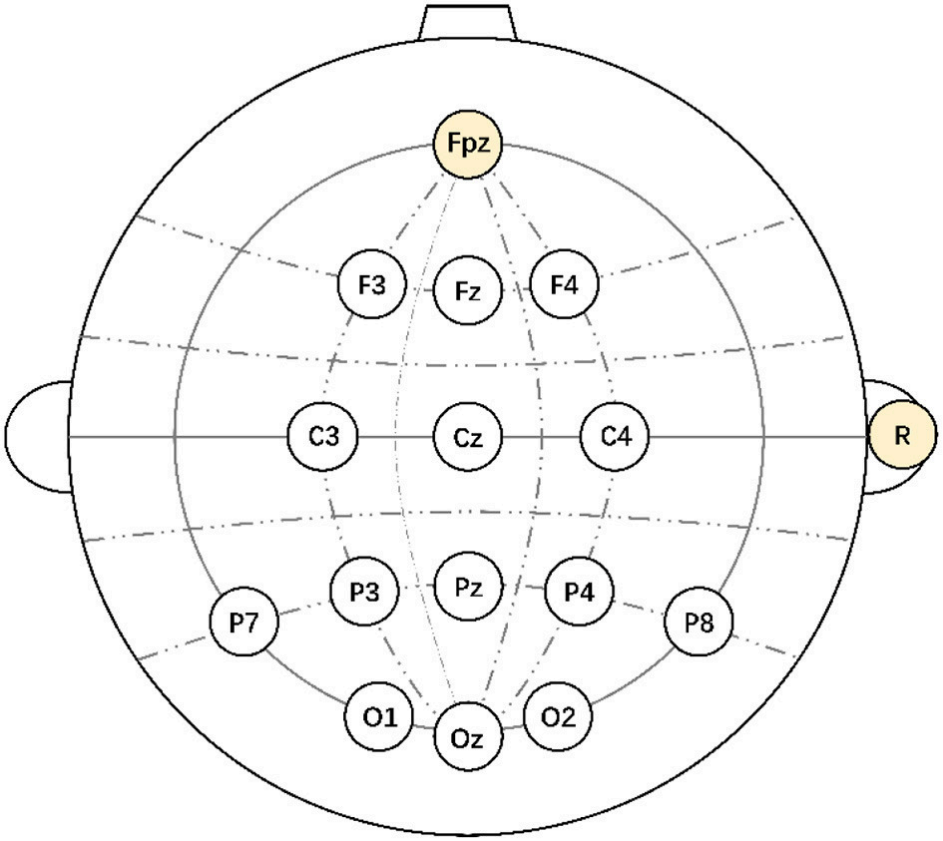
The electrodes selected from the 10–20 system.

In this study, the EEG signals were recorded by g.USBamp and 32-channel g.EEGcap (Guger Technologies, Graz, Austria). The amplifier was set with a sample rate of 256 Hz, a sensitivity line of 100 μV, a band-pass filter from 0.5 to 30 Hz, a notch filter at 50 Hz to remove AC artifacts, and impedances below 10 kΩ. All 14 electrodes selected from the 10–20 international system were F3, Fz, F4, C3, Cz, C4, P7, P3, Pz, P4, P8, O1, Oz, and O2, which were referenced at right mastoid and grounded at FPz (Figure 3).

### Feature Extraction and Classification

After the offline data acquisition for each subject, feature extraction and classification were performed to build a personal model for his or her online session later. In terms of filtering, a third-order Butterworth filter with a band pass from 1 to 30 Hz was applied to raw EEG data. Then, according to the labels attached to every flash (which were simultaneously made during the data acquisition), the 100-ms pre-stimulus (flash) and the 800-ms post-stimulus data segments (altogether 900-ms data segment) were selected. Moreover, the latter 800 ms was reserved after baseline correction by means of the former 100 ms. Thereform, a three-dimensional matrix was constructed by the factors of channels, sampling rate, and trials realized for one subject. As for downsampling, the second dimension (related to time shaft) of the matrix was downsampled to 36 Hz, instead of the original sample rate of 256 Hz. Therefore, the feature vector with 14 channels × 29 time points was accessible for the classifier.

Here, we adopted the Bayesian linear discriminant analysis (BLDA), which was first developed by Hoffmann et al. (2008) and successfully applied to a P300-BCI system to classify EEG data, because of its capability to better overcome the overfitting of high-dimensional data or data containing noise. Moreover, this method is relatively efficient in the ERP-BCI system (Chen et al., 2015). Then, 16-fold cross-validation was performed after model building, so that the scores of each flash can be achieved, and the target flash can obtain the highest score among the eight.

**Figure 4:**
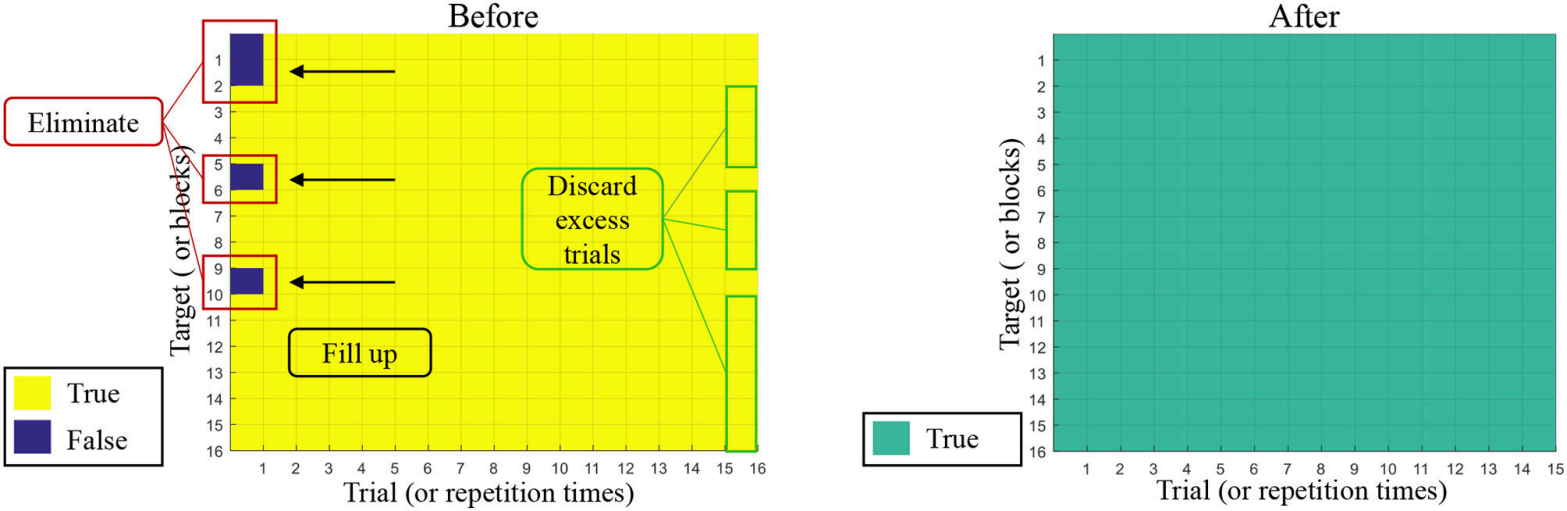
Example for the error trials before and after model modification.

To improve the model, we applied a trial selection method to help eliminate the error trials in offline data. For example, one block needs to be counted for 16 times in one run (see Figure 2). In Figure 4, we demonstrated the whole process. If the first trial was recognized as false according to the classifier, the “first” would be removed (like the red frame in the left panel of Figure 4), and the 15 remaining trials would fill up; however, not all the blocks enjoyed 16 times of repetition after eliminating, so on account of the integrity and uniformity, we discarded some trials in the green frame and kept all blocks with 15 repetition times (see the right panel of Figure 4). In this case, we eliminated the distraction brought by the new start of a target fixation to some extent. This modification was only executed once, considering the sufficiency of data used to perform the subsequent overlapping averaging process. Then, the rest of the trials were sent to the classifier again to rebuild a model for favorable performance.

### Online Strategy

After achieving the model developed on the basis of offline datasets, the online real-time feedback could be presented smoothly to the subject every time as one block's recognition was completed. However, it took fewer trials than an epoch did in offline session, because the system judged whether the last two successive results were the same in every block recognition. If so, the process of trials for the block would be stopped, and the last result would be shown as the feedback. Otherwise, the maximum trials of one block, which was set to 16, would be performed. In this way, the feedback of each block was printed successively on the screen until the 16 blocks were completely recognized.

### Data Analysis and Statistics

Two important performance indexes are accuracy and information transfer rate (ITR), which were used to evaluate a BCI system. The latter one can be calculated as follows:

(1) $$B=\{\begin{array}{cc} \log_{2}N+Acc\cdot \log_{2}Acc+\left(1-Acc\right)\cdot \log_{2}\left(\frac{1-Acc}{N-1}\right) & 0<Acc<1\\ \log_{2}N+Acc\cdot \log_{2}Acc & Acc=1 \end{array}$$

(2) $$ITR=\frac{B\cdot 60}{t}$$

In formula 1, *N* denotes the possible choices in one trial, and whereas every choice shares the equal possibility to be lighted, here *N* = 8; *Acc* represents the classification accuracy. In formula 2, *t* is the time cost for operating the trials, and *ITR* (bit/min) can be achieved through calculation.

In terms of statistics, all the variables were first tested under Ryan–Joiner test (R-J test), which is similar to the Shapiro–Wilk test, for normal distribution. Then, repeated-measures ANOVA (rm-ANOVA) was applied to test the significance brought by the color factor. However, before RM-ANOVA, Mauchly's sphericity test was executed, and if unsatisfactory, Greenhouse–Geisser correction would be chosen to revise degree of freedom. Finally, Bonferroni correction was implemented in *post hoc* comparison. The significance level was α = 0.05 after Bonferroni correction.

### Color Contrast Calculation

Li et al. (2014) investigated the effects of luminosity contrast on BCI performance. It was reported that higher classification accuracy was achieved by a high-luminosity contrast; higher amplitude and shorter latency of VEP P300 were also released by the high-luminosity contrast stimulus. The following were the calculation formulas of luminosity contrast mentioned in Li's study:

(3) L=0.2126*R+0.7152*G+0.0722*B

(4) $$X=\{\begin{array}{ll} \left(\left(X_{sRGB}+0.0550\right)/1.055\right)ˆ2.4 & X_{sRGB}>0.03928\\ \;X_{sRGB}/12.92 & X_{sRGB}\geq 0.03928 \end{array}$$

The *X* above can be R or G or B.

(5) $$\begin{array}{l} R_{sRGB}=R_{8bit}/255\\ G_{sRGB}=G_{8bit}/255\\ B_{sRGB}=B_{8bit}/255 \end{array}$$

The ratio between the display color (L1) and the background color (L2) is

(6) $$\text{Luminosity Contrast Ratio}=\frac{L_{1}+0.05}{L_{2}+0.05}$$

In this work, we calculated the corresponding ratio for the three stimuli under white background according to the formulas above and discussed the results in the section Layout of the Stimulus.

## Results

### ERP Analysis

**Figure 5:**
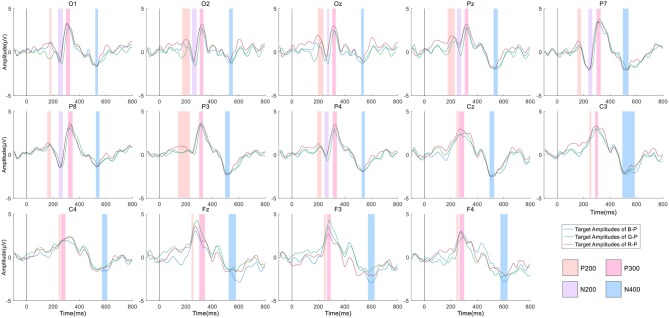
Grand averaged evoked related potential (ERP) waveforms of targets across all 12 subjects from three paradigms overall 14 electrodes. *Note:* Four kinds of ERP signals (e.g., P2, N2, P3, N4) were demonstrated with different backgrounds in each plot of the electrode if existing.

Figure 5 shows the grand averaged ERP waveforms over 14 channels with three curves representing three different color types of stimulus in a single-channel plot. Four kinds of colors were shadowed behind the neighborhood of peak point, with the rule that the minimum and the maximum of three peak points (latency) would be selected, and the range would be formed [min −10 ms, max +10 ms]. Such rules were also feasible for the condition that only one or two curves displayed the desired signal, whereas the rest did not.

**Figure 6:**
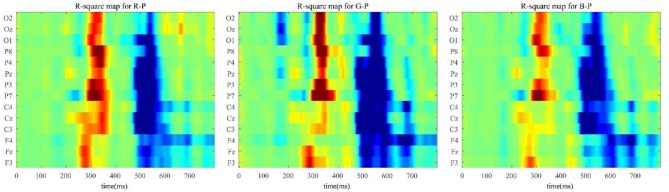
R-squared value maps of three paradigms throughout all 14 channels for S1–S12 subjects for discrimination between the target and the non-target.

Figure 6 illustrates the discrimination between the target and the non-target over all sites from the three paradigms. We applied a time window with 0–800 ms after a stimulus and considered the target and non-target ERP segments as the inputs of the calculation shown below to obtain the R-squared values.

(7) $$r{\left(x\right)}^{2}={\left(\frac{\sqrt{N_{1}N_{0}}}{N_{1}+N_{0}}\cdot \frac{mean\left\{x|y=1\right\}-mean\left\{x|y=0\right\}}{std\left\{x|y=1,0\right\}}\right)}^{2}$$

In formula 7, *x* represents the value; *y* can be “1” standing for the target samples, whereas “0” for the non-target ones; and *N*_1_ and *N*_0_ are the corresponding numbers of the groups. In Figure 6, the polar color turned darker as discrimination went more obvious between the two.

#### P2

P2 peak is typically evoked following N100 in visual ERP-based BCI and varies between 150 and 275 ms. P2 is related to visual search, attention, and memory (Freunberger et al., 2007). In this study, we explored P2 in parieto-occipital areas of the scalp. Through statistical analysis, the stimulus color significantly affected the P2 peak latency at electrodes of Oz [*F*_(2, 22)_ = 8.762, *p* < 0.01]. Then, comparison within groups indicated that G-P's P2 latency was observed significantly longer than that of B-P at Oz (*p* < 0.01).

#### N2

**Figure 7:**
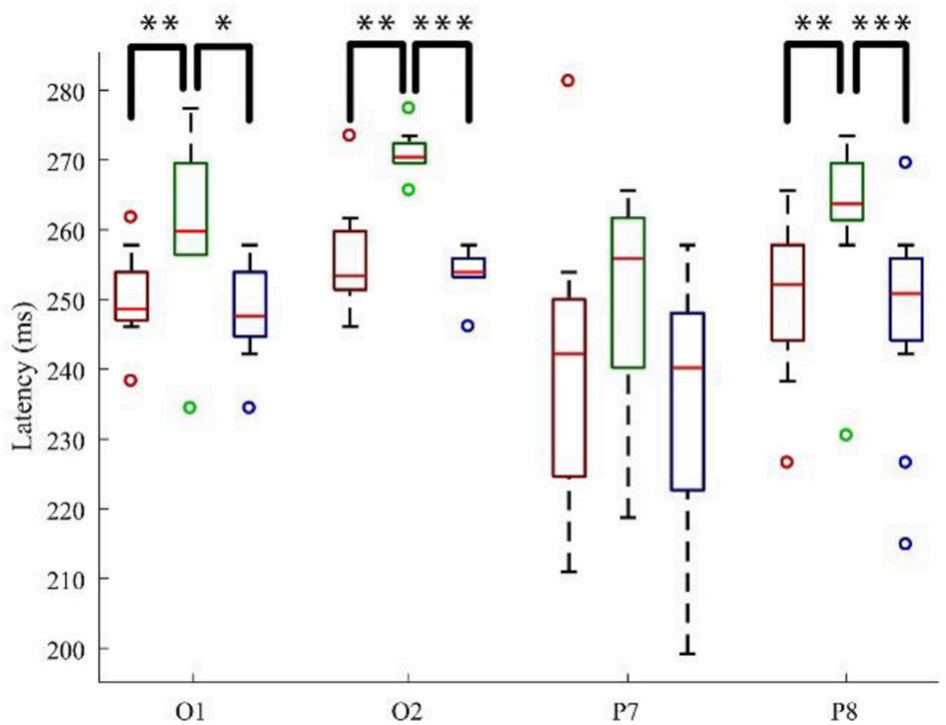
The N200 latency boxplot with significance at four sites. *Note:* the label “*” means the significance of two groups is *p* < 0.05; meanwhile, **p < 0.01 and ***p < 0.001; the color of the box indicates the corresponding paradigm.

In Figure 7, the significance of the N2 peak latency was revealed at O1 [*F*_(2, 22)_ = 11.672, *p* < 0.01; G-P > R-P: *p* < 0.01, G-P > B-P: *p* < 0.05], at O2 [*F*_(2, 22)_ = 30.078, *p* < 0.01; G-P > R-P: *p* < 0.01, G-P > B-P: *p* < 0.001], and at P8 [*F*_(2, 22)_ = 17.870, *p* < 0.01; G-P > R-P: *p* < 0.01, G-P > B-P: *p* < 0.001]. Thus, the N2 peak evoked by G-P was later than that for R-P and B-P significantly at electrodes O1, O2, and P8, respectively. However, no significance has been detected either in tests of within-subjects effects or in *post hoc* multiple comparisons in terms of N2 amplitude.

### Accuracy and Bit Rate of Brain–Computer Interface

**Figure 8:**
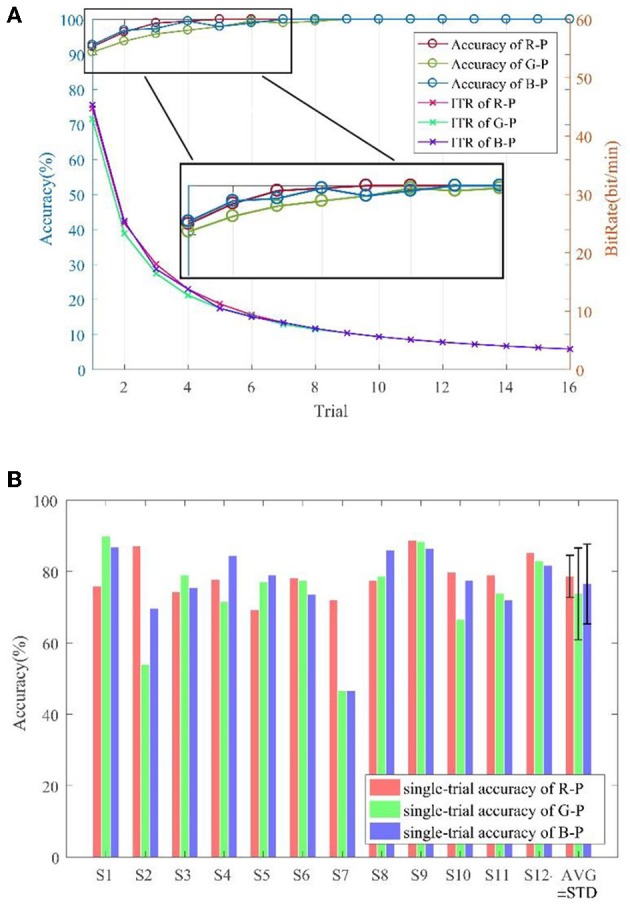
Offline accuracy and bit rate analysis. **(A)** Overlapping average per trial. **(B)** Single-trial per subject.

Figure 8A displays the offline accuracy and bit rate, which was averaged over 12 subjects and overlapping by trials. R-P yielded a better offline performance depending on the highest offline accuracy and the least trials to reach 100%. Furthermore, Figure 8B depicts the single trial offline classification accuracy, but no significant difference was found.

Table 2 lists the online performance of 12 subjects in detail, and *p*-value was tested among the three paradigms for three indexes [i.e., accuracy (%), ITR (bit/min), and AVT] closely behind. However, *p*-value shows significance in accuracy and ITR between R-P and G-P.

**Table 2:** Online accuracy and bit rate analysis.

	**Accuracy (%)**			**Bit rate (bit/min)**			**AVT**		
	**R-P**	**G-P**	**B-P**	**R-P**	**G-P**	**B-P**	**R-P**	**G-P**	**B-P**
S1	100.00	100.00	100.00	24.97	25.71	25.00	2.19	2.19	2.25
S2	100.00	87.50	62.50	27.27	17.07	7.09	2.06	2.31	2.63
S3	100.00	87.50	100.00	23.68	16.20	25.00	2.38	2.44	2.25
S4	100.00	93.75	93.75	23.68	20.17	20.73	2.38	2.31	2.25
S5	100.00	93.75	100.00	24.32	20.17	27.27	2.31	2.31	2.06
S6	100.00	93.75	93.75	26.47	20.73	20.17	2.13	2.25	2.31
S7	100.00	87.50	81.25	26.47	16.62	13.67	2.13	2.38	2.44
S8	100.00	93.75	100.00	25.71	21.32	26.47	2.19	2.19	2.13
S9	100.00	93.75	100.00	24.32	19.13	24.32	2.31	2.44	2.31
S10	93.75	87.50	100.00	20.17	15.79	25.71	2.31	2.50	2.19
S11	87.50	93.75	93.75	16.20	20.73	20.17	2.44	2.25	2.31
S12	100.00	100.00	93.75	26.47	26.47	21.32	2.13	2.13	2.19
AVG	**98.44**	92.71	93.23	**24.14**	20.01	21.41	**2.25**	2.31	2.28
STD	3.72	4.29	10.66	3.01	3.29	5.64	0.12	0.11	0.14
*p*	**R-P vs. G-P**	G-P vs. B-P	R-P vs. B-P	**R-P vs. G-P**	G-P vs. B-P	R-P vs. B-P	R-P vs. G-P	G-P vs. B-P	R-P vs. B-P
	**0.014**	1.000	0.498	**0.019**	1.000	0.693	0.401	1.000	1.000

*AVT refers to an average number of trials consumed to output the feedback; Bonferroni correction has been applied. The bold values mean the highest accuracy, bit rate, and the least repetition times among those 3 colors, and in the row of p-value, bold one was to highlight the pair which achieved significance*.

### Effects by Model Modification

**Figure 9:**
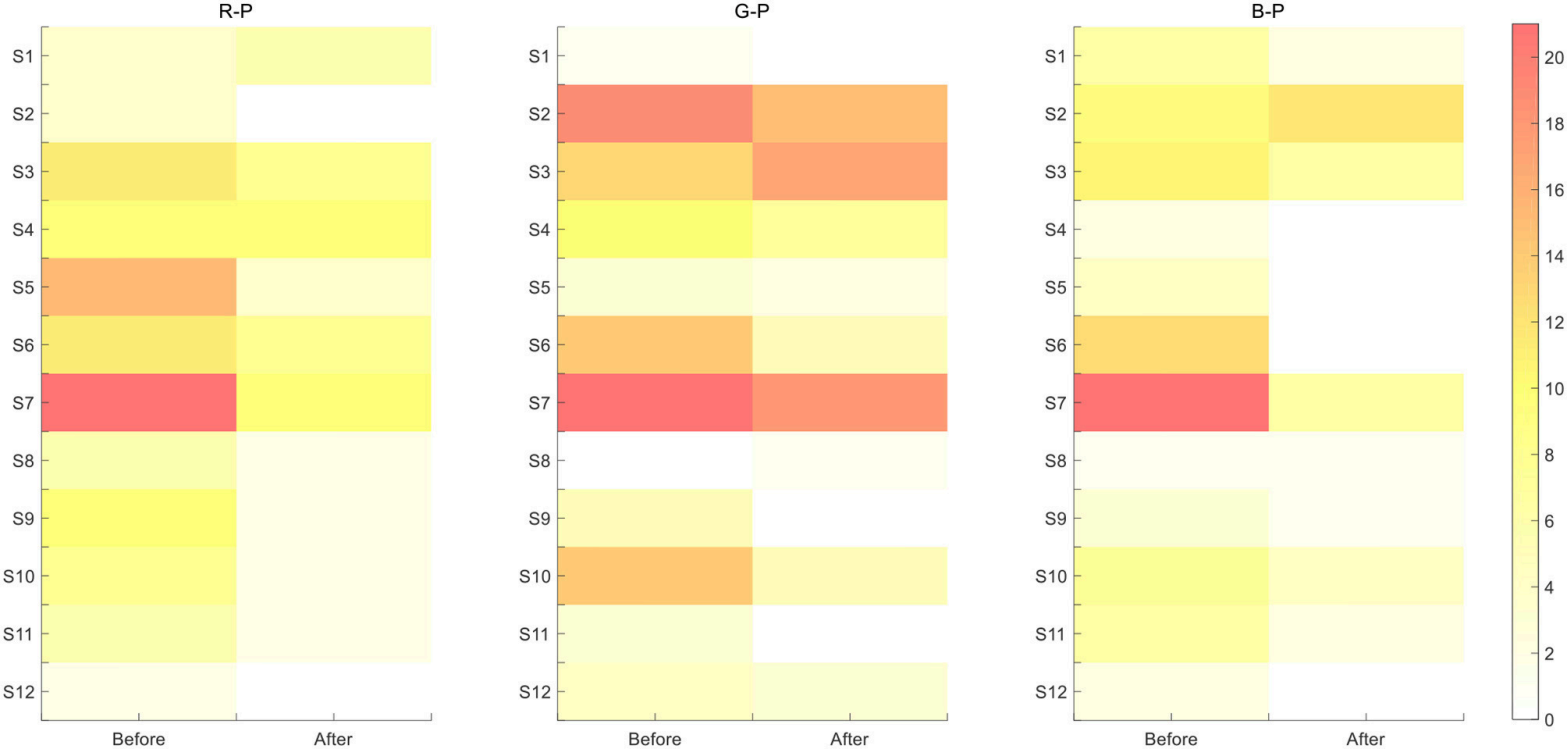
The error trials before and after modification of the model. *Note:* the number implies the error trials happened for one subject in one paradigm and the overall sum of trials was 240 (16 targets × 15 times); the statistical results in this section were all corrected by Bonferroni correction.

By utilizing the method mentioned in the section Feature Extraction and Classification, the error trials changed, as displayed in Figure 9. Through two-way RM-ANOVA with the factors of method (before and after model modification) and stimulus color, significance was found in the factor of color [*F*_(2, 22)_ = 4.942, *p* < 0.05] and method [*F*_(1, 11)_ = 21.868, *p* < 0.01] while it was not found in the interaction of the two factors [*F*_(2, 22)_ = 0.979, *p* > 0.05]. In *post hoc* of model modification, error trials were significantly reduced (*p* < 0.01). Meanwhile, the sum of error trials for subjects after the modification was significantly reduced [*F*_(1, 11)_ = 21.868, *p* < 0.01] as well. Thus, the efficiency of the model modification method in this work was proved.

Moreover, although stimulus color significantly influenced the error trials before modification [*F*_(2, 22)_ = 4.585, *p* < 0.05] and after it [*F*_(2, 22)_ = 4.040, *p* < 0.05], no significance was found in *post hoc* in error trials either before modification or after it.

### Effects by the Layout

As mentioned in the section Effects by Model Modification, the layout of the pattern may also influence the offline accuracy. Through two-way RM-ANOVA, we found that the interaction of the two factors was significant [*F*_(2, 22)_ = 4.424, *p* < 0.05]. Therefore, we shifted the two-way RM-ANOVA to one-way RM-ANOVA to detect the simple effect of each factor. The layout factor affected error trials significantly in G-P [*F*_(1, 11)_ = 6.289, *p* < 0.05] and in the sum [*F*_(1, 11)_ = 5.482, *p* < 0.05].

**Figure 10:**
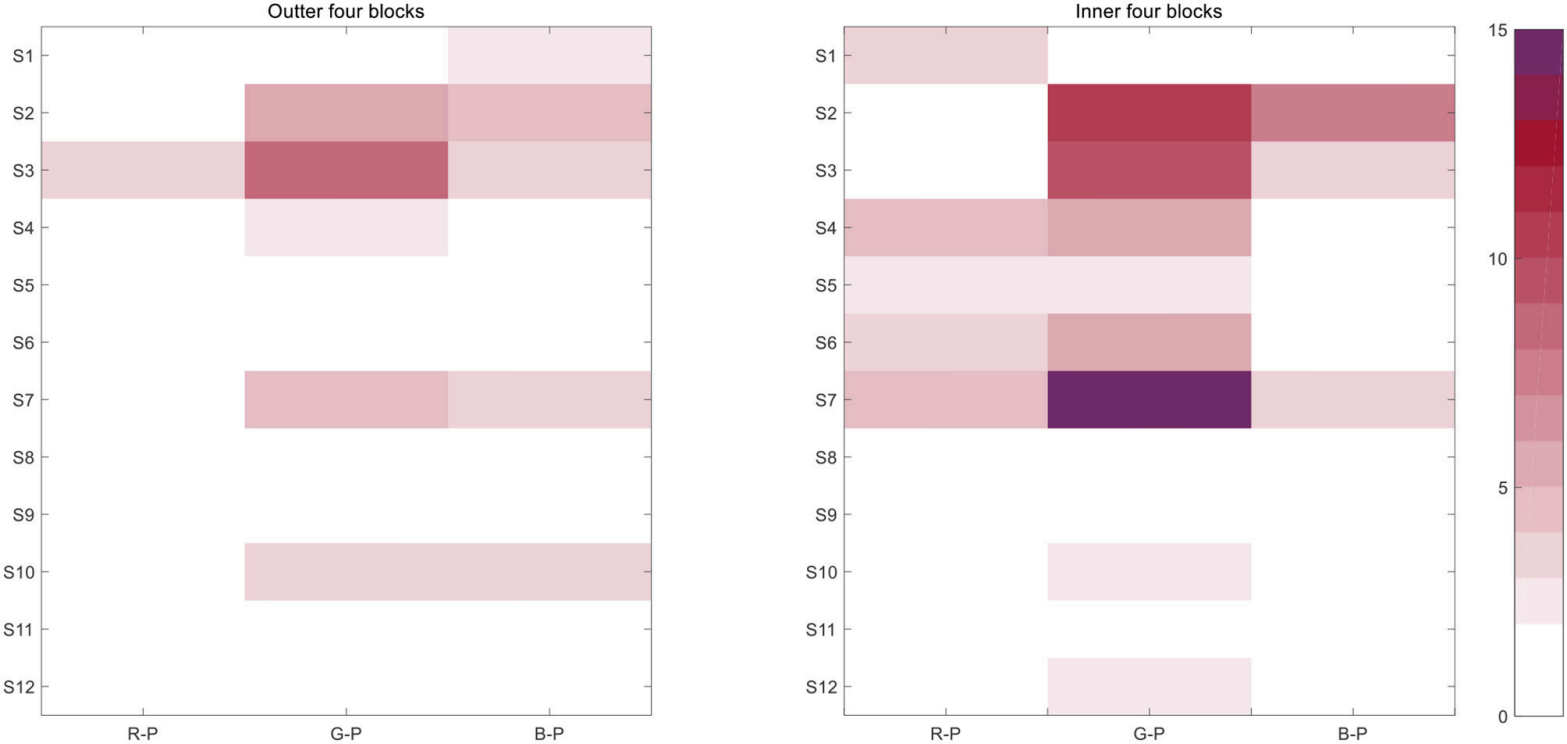
The error trials contributed by outer and inner blocks. *Note:* the number implies the error trials happened for one subject in one paradigm and the overall sum of trials was 240 (16 targets × 15 times); the statistical results in this section were all corrected by Bonferroni correction.

When color acted as the factor, significance was only observed before modification [*F*_(2, 22)_ = 4.545, *p* < 0.05; G-P > B-P: *p* < 0.05). Nevertheless, the four inner blocks produced more error trials than the outer ones, and the difference was significant (Figure 10).

The sum of offline error trials was 48 in the four outer blocks through the three paradigms, and it counted to 85 in the inner ones. Specifically, 8,640 times [16 targets × (16–1) times × 12 subjects × 3 paradigms] was counted by 12 subjects during the three paradigms; thus, 0.56% error rate occurred in the four outer blocks, and 0.98% occurred in the inner ones.

### Subjects' Feedback

Subjects were asked to evaluate the tiredness of each paradigm by scores (1: few; 2: medium; 3: many). To specify the differences, we also applied Friedman test to investigate the differences on scores. Friedman test as a type of non-parametric test was appropriate for those correlated samples (Table 3). However, no significance was found toward tiredness (χ^2^ = 1.267, *p* > 0.05).

**Table 3:** Subjects' feedback to each paradigm.

	**S1**	**S2**	**S3**	**S4**	**S5**	**S6**	**S7**	**S8**	**S9**	**S10**	**S11**	**S12**
R-P	1	1	3	1	2	2	1	2	2	2	3	2
G-P	1	1	1	2	3	2	2	1	1	2	2	1
B-P	2	1	2	1	1	2	2	2	1	2	1	1

*A score of “1” means a little tired, “2” means medium, and “3” means quite a lot*.

## Discussion

The present study mainly focused on the effect of chromatic stimulus on the performance of an ERP-based BCI and discussed several related problems stated as follows: (1) the influence on the offline error trials brought by the layout and the relationship between the layout and adjacency distraction, (2) the availability of the conclusion (Li et al., 2014) applied to the present study that better performance (including higher accuracy, higher amplitude, and shorter latency) occurred in a high-luminosity contrast, and (3) the observation of the ERP components' waveforms in this work.

### Performance

As for performance, classification accuracy and ITR were the main indexes to evaluate the performance of a BCI system. The online result (Table 2) depicted that the highest online averaged accuracy was obtained by R-P with 98.44%, higher than 92.71% by G-P, and 93.23% by B-P. In addition, significance was found between R-P and G-P both over online accuracy (*p* < 0.05) and ITR (*p* < 0.05), under the circumstance that all subjects were divided into three groups to experience the three paradigms in three kinds of order. Thus, the effects by the order of process were eliminated.

To explain the result, some literature in psychology may help. It was found that longer-wavelength colors including red are considered as arousing or warm, whereas colors with a shorter wavelength like green and blue are associated with relaxing and cool (Nakshian, 1964). For one color as stimulus to be experienced lasting for 40 min at least in our experiment, the color of stimulus when flashing may exert some psychological hint to motivate or cool down the emotion of subjects to some degree. Some psychological experiments found that red can promote performance on some virtual target-shooting task (Sorokowski and Szmajke, 2011). They reported that the participants were able to hit red moving objects significantly better than blue and black objects, which was much relevant to our study in both stimulus color and the conclusion. On the side of biology, it was known that objects' information of color was described to be processed in visual area V4 of the human brain (Dubner and Zeki, 1971) and the cones in human's eyes have different light sensitivity to red, green, and blue light. This paper's result may give some evidence or reference to help related biological research.

In related studies, the green/blue flicker paradigm achieved an 80.60% online classification accuracy (Takano et al., 2009). The paradigm that set a green familiar face as stimulus yielded an 86.1% online accuracy on average (Li et al., 2015). An SSVEP-BCI utilizing red, green, blue, and violet as stimuli showed that the violet one gained the highest accuracy of 94.38%, and the red one obtained 90.21% in wheelchair control application (Singla et al., 2013). Hence, the novel BCI with chromatic stimulus is consistent, efficient, and practicable, as judged by extracting consistent ERP wave features and outstanding mean accuracy over 90% online experiments for all 12 subjects.

### Layout of the Stimulus

In this work, we applied a novel layout paradigm with chromatic stimulus flashing in blocks on the basis of SCP. The benefits of this design lie in two parts. One is the problem of double flash. Considering that eight blocks randomly flashed once in one trial, and the SOA of one flash is 400 ms, a single target cannot possibly flash twice in a time interval shorter than 800 ms. The other is adjacency distraction. As shown in the section Effects by the Layout, the position indeed influenced the error trials in offline sessions significantly, but the ratios it caused were 0.56% for the outer blocks and 0.98% for the inner blocks, thereby indicating a comparatively minor aspect in terms of the whole situation, especially after model modification.

### Color Contrast

As mentioned in the section Color Contrast Calculation, the color contrast ratio was 2.61:1 for R-P, 1.29:1 for G-P, and 3.66:1 for B-P with a white background. In previous literature (Nam et al., 2010; Li et al., 2014), all of the values of RGB channels remained equal, and the groups for contrast were limited to two. However, when the comparison groups of stimulus color increased to three in the present study, several previous results did not show similarity with the trend. In P300 waveform, no satisfactory significance was shown in the P300 amplitude of three paradigms within subjects at Pz, inconsistent with the trend in the literature. For online accuracy, a higher averaged accuracy was obtained by R-P, followed by B-P and G-P, as shown in Table 2; hence, G-P had the lowest color contrast ranked at the bottom, whereas the results of R-P and G-P cannot be satisfied by that observation. Moreover, the relationship between color contrast and accuracy is not linear.

### ERP Component

Visual stimulus features such as color are processed in the ventral stream of visual pathways over the occipitotemporal areas of the brain (Corbetta et al., 1991; Merigan and Maunsell, 1993).

P2 peak waveform features in the present study resulted in obtaining a longer latency in G-P at Oz. The oddball paradigm is one primary way to evoke P2, and its amplitude can be enhanced to the targets (Ferreira-Santos et al., 2012). However, in a visual search paradigm, more specific research has been performed on stimulus features (e.g., color, size, and orientation) to explore the mechanisms for feature detection in the brain (Luck and Hillyard, 1994). Thus, the findings in the present work are relatively supplemented in this area.

N2, which is an endogenous component similar to P300, corresponds to visual attention or degree of attention. In the present study, the N2 latency from G-P was significantly longer than that of the two other paradigms within all subjects. This result was caused by a shorter latency shown in high color contrast, whereas a longer latency was shown in low color contrast (Li et al., 2014). Here, “green” obtained the lowest value in color contrast at the white background.

Meanwhile, P300 and N4 failed to exhibit significance either in amplitude or in latency. As shown in Figure 5, the three grand averaged curves were relatively close to each other under the color shadows of P300 and N4 waveforms, thereby indicating that P300 and N4 were not sensitive to different stimulus colors in this work.

## Conclusion

The color of stimulus out of RGB could achieve the best performance in an ERP-based BCI by designing a novel layout in a single-character pattern. In detail, R-P yielded the highest online averaged accuracy and the fastest ITR among the three; G-P displayed a longer latency in the ERP waveforms of P2 and N2. Moreover, the eight blocks in the paradigm can be replaced with control commands or be applied to psychological attention estimation. Further investigation will be performed on the neural mechanism of our experimental results. Besides, further improvement may focus on the algorithm improvement, enhancement of ITR, and fatigue supervision (e.g., heart rate and body temperature).

## Ethics Statement

This work was approved by Shanghai Xuhui Central Hospital Committee, SOP-IEC-033-01.0-AF02. This study was carried out in accordance with the recommendations of name of guidelines, name of committee with written informed consent from all subjects. All subjects gave written informed consent in accordance with the Declaration of Helsinki. The protocol was approved by the name of committee.

## Author Contributions

MG designed the concept of the manuscript, designed the whole experiment, and collected the original data set. All authors contributed to manuscript revision and read and approved the submitted version.

### Conflict of Interest Statement

The authors declare that the research was conducted in the absence of any commercial or financial relationships that could be construed as a potential conflict of interest.
